# Supplementary material for: The Influence of Subjective Socioeconomic Status on Life Satisfaction: The Chain Mediating Role of Social Equity and Social Trust
Source: Int J Environ Res Public Health. 2022 Nov 25;19(23):15652. doi: 10.3390/ijerph192315652 (PMC9738263; doi:10.3390/ijerph192315652)
Supplement: Supplementary file 1 [file ijerph-19-15652-s001.zip › ijerph-1996836-supplementary.pdf]

Table S1. Sociodemographic statistics (N=17,217).

Variable	Categories	Frequency	Valid Percentage
Gender	Male	7683	44.6%
	Female	9534	55.4%
Marital Status	Unmarried	2238	13.0%
	First marriage	13181	76.6%
	Remarry	611	3.5%
	Divorce	443	2.6%
	Wid	695	4.0%
	Live together	49	0.3%
Residence	Urban	5099	29.6%
	Urban-rural fringe area	563	5.0%
	town	2738	15.9%
	Township combined area	960	5.6%
	Special area	89	0.5%
	countryside	429	2.5%
	village	7039	40.9%
Age	17-24	1697	9.9%
	25-30	1610	9.4%
	31-40	2861	16.6%

	41-50	3789	22.0%
	51-60	3977	23.1%
	61-70	3281	19.1%
Educational attainment	Uneducated	1500	8.7%
	Primary	3713	21.6%
	Junior high	5475	31.8%
	Senior high	2321	13.5%
	Technical secondary	811	4.7%
	Professional high	154	0.9%
	Junior college	1479	8.6%
	Undergraduate college	1597	9.3%
	Graduate student	142	0.8%
	Others	25	0.1%
Subjective socioeconomic status	1	4591	26.7%
	2	5111	29.7%
	3	6538	38.0%
	4	871	5.1%
	5	106	0.6%

The survey included a number of indicators of social equity, such as the college admissions process, the actual political freedoms citizens actually enjoy, the administration of justice and law enforcement, public health care, job and employment opportunities, wealth and income distribution, social security benefits like old-age care, and rights and treatment between urban and rural areas. We performed a factor analysis

on these eight difficulties and discovered that just one factor could be evaluated. We refer to this element as social fairness. The KMO statistic was 0.877, and the Bartlett test result was less than 0.05, which is adequate for factor analysis, according to the KMO and Bartlett tests.

Table S2. KMO and Bartlett test results.

KMO sampling suitability quantity		0.877
Bartlett Sphericity Test	Approximate chi-square	36230.077
	df	28
	Sig.	0.000

The results of the factor analysis are shown in Table 2

Table S3. Results of factor analysis.

Ingredients	Initial eigenvalues			Extracting the sum of squares and loading		
	Summation	Variance %	Cumulation %	Summation	Variance %	Cumulation %
1	3.565	44.566	44.566	3.565	44.566	44.566
2	.911	11.386	55.952			
3	.717	8.960	64.911			
4	.701	8.757	73.668			
5	.629	7.865	81.533			
6	.535	6.688	88.220			
7	.473	5.917	94.137			
8	.469	5.863	100.000			

Extraction method: Main ingredient analysis.

We determined the weight of different subjects using factor analysis. The fairness of the college admissions process accounted for 10% of these, while political rights actually exercised by citizens accounted for 13%, the administration of justice and law enforcement accounted for 13%, the fair share of public health care accounted for 13%, fair work and employment opportunities accounted for 13%, the fair distribution of wealth and income accounted for 13%, and between urban and rural areas accounted for 12%. Rights are treated fairly in 12% of cases.
